# IL-17 Inhibits Chondrogenic Differentiation of Human Mesenchymal Stem Cells

**DOI:** 10.1371/journal.pone.0079463

**Published:** 2013-11-15

**Authors:** Masahiro Kondo, Kunihiro Yamaoka, Koshiro Sonomoto, Shunsuke Fukuyo, Koichi Oshita, Yosuke Okada, Yoshiya Tanaka

**Affiliations:** 1 The First Department of Internal Medicine, University of Occupational and Environmental Health, Yahatanishi-ku, Kitakyushu, Fukuoka, Japan; 2 Pharmacology Research Laboratories I, Research Division, Mitsubishi Tanabe Pharma Corporation, Aoba-ku, Yokohama, Kanagawa, Japan; Instituto de Engenharia Biomédica, University of Porto, Portugal

## Abstract

**Objective:**

Mesenchymal stem cells (MSCs) can differentiate into cells of mesenchymal lineages, such as osteoblasts and chondrocytes. Here we investigated the effects of IL-17, a key cytokine in chronic inflammation, on chondrogenic differentiation of human MSCs.

**Methods:**

Human bone marrow MSCs were pellet cultured in chondrogenic induction medium containing TGF-β3. Chondrogenic differentiation was detected by cartilage matrix accumulation and chondrogenic marker gene expression.

**Results:**

Over-expression of cartilage matrix and chondrogenic marker genes was noted in chondrogenic cultures, but was inhibited by IL-17 in a dose-dependent manner. Expression and phosphorylation of SOX9, the master transcription factor for chondrogenesis, were induced within 2 days and phosphorylated SOX9 was stably maintained until day 21. IL-17 did not alter total SOX9 expression, but significantly suppressed SOX9 phosphorylation in a dose-dependent manner. At day 7, IL-17 also suppressed the activity of cAMP-dependent protein kinase A (PKA), which is known to phosphorylate SOX9. H89, a selective PKA inhibitor, also suppressed SOX9 phosphorylation, expression of chondrogenic markers and cartilage matrix, and also decreased chondrogenesis.

**Conclusions:**

IL-17 inhibited chondrogenesis of human MSCs through the suppression of PKA activity and SOX9 phosphorylation. These results suggest that chondrogenic differentiation of MSCs can be inhibited by a mechanism triggered by IL-17 under chronic inflammation.

## Introduction

Chondrocytes were considered as the only cell type that exists in the articular cartilage until recently. Due to the limited regenerative ability of chondrocytes, cartilage defects in patients with rheumatoid arthritis (RA), osteoarthritis (OA), and trauma are irreversible and cartilage repair is considered difficult. However, some reports have characterized mesenchymal stem cells (MSCs) in the articular cartilage as chondrocyte progenitor cells [1]–[3]. MSCs are multipotent cells capable of differentiation into osteoblasts and chondrocytes, and can be easily obtained from mesodermal tissues, such as bone marrow and adipose tissue [4]. We reported previously that MSCs can effectively differentiate into osteoblasts in the presence of IL-1β through the non-canonical WNT5A/ROR2 signaling pathway [5]. In addition, MSCs produce high amounts of osteoprotegerin, a decoy receptor for receptor activator of nuclear factor kappa-B ligand (RANKL), and efficiently suppressed osteoclast differentiation, highlighting the potential importance of MSCs in joint repair treatments [6]. Furthermore, several studies have shown that implanted MSCs can differentiate into chondrocyte-like cells *in vivo* [7], [8]. Given the finding that MSCs exist in articular cartilage, it is conceivable that MSCs contribute to the maintenance of cartilage homeostasis and, moreover, possess an intrinsic ability to repair cartilage defects. TNF-α has been reported to inhibit chondrogenic differentiation of human MSCs [9] and, correspondingly, the repair of articular cartilage can be observed in some RA patients treated with TNF inhibitors [10].

The relationship between chronic inflammation and bone or cartilage metabolism has been gaining attention in recent years. IL-17A (hereafter referred to as IL-17), a key player in chronic inflammation, is characterized as a bone metabolism-related cytokine [11]. In animal models that have employed strategies to increase expression, neutralize activity, or delete IL-17, IL-17 plays a distinct pathogenic role in inflammatory arthritis [12]–[14]. In human studies, high concentrations of IL-17 have been found in the synovial fluid of patients with RA [15]–[17] and IL-17-producing CD4^+^ T lymphocytes (Th17 cells) have been detected in RA synovial membranes [18]. In cartilage, IL-17 induces cartilage matrix breakdown by increasing the expression of matrix metalloproteinases (MMPs) in synoviocytes and chondrocytes [19], [20], as well as apoptosis of chondrocytes, which is thought to contribute to cartilage destruction [21]. However, there are only few reports on the effects of inflammatory cytokines on chondrocyte differentiation. In particular, inflammatory cytokines are concentrated in the joints of RA patients and are predicted to inhibit chondrogenic differentiation of MSCs. However, the specific effects of IL-17 on chondrogenesis have not been reported.

Transforming growth factor beta (TGF-β) is a pleiotropic growth factor that is involved in multiple cellular processes and is widely known to promote chondrogenic differentiation [4]. Among the various TGF-β signaling pathways, SMADs, the downstream transcription factors, play a distinct role in regulation of cartilage related genes [22]. On the other hand, SRY-type HMG box9 (SOX9) has been identified as a master transcription factor in chondrogenic differentiation [23]–[25]. Transcriptional activity of SOX9 is regulated by not only its expression level but also several post-translational modifications including phosphorylation. Cyclic adenosine monophosphate (cAMP)-dependent protein kinase A (PKA) is a key kinase known to phosphorylate SOX9 [26], [27]. However, the effect of IL-17 on these TGF-SMAD pathway and PKA-SOX9 axis remains unclear.

Here we evaluated the effects of IL-17 on chondrogenic differentiation of human MSCs utilizing an *in vitro* pellet culture system with TGF-β3. The results showed that IL-17 inhibited chondrogenesis through a mechanism involving PKA and SOX9 activity. These findings have important implications for the design of clinically effective cartilage repair therapies.

## Materials and Methods

### Cell Culture

Human MSCs were purchased from Lonza (Walkersville, MD). Multipotency was confirmed by differentiation of MSCs into osteoblasts, chondrocytes, and adipocytes. Cell surface markers were positive for CD29, CD44, CD105, and CD166 and negative for CD14, CD34, and CD45. Cells were cultured following the instructions recommended by the manufacturer. Cells were cultured in MSC growth medium (MSCGM) (Lonza) at 37°C in a 5% CO_2_ atmosphere and maintained at subconfluence to prevent spontaneous differentiation. Cells from passage 2–4 were used in this study.

### Chondrogenesis and Cell Treatment

Human MSCs at subconfluent conditions were trypsinized and aliquots of 2×10^5^ cells per well were added to an ultra-low attachment surface, round-bottom 96-well plate (Corning, New York, NY), and the plate was spun at 400×*g* for 5 min. For differentiation into chondrocytes, cells were cultured in a commercialized chondrogenic induction medium (hMSC Differentiation BulletKit-chondrogenic, Lonza) in the absence or presence of 10 ng/mL recombinant human TGF-β3 (Lonza). The cell pellets formed free-floating aggregates within the first 24 h. To analyze the effects of inflammatory cytokines or a kinase inhibitor, recombinant human IL-17A (Pepro Tech EC, London, UK), TNF-α (R&D Systems, Minneapolis, MN), IL-1β (RELIATech, San Pablo, CA), or the PKA inhibitor H89 (Enzo Life Sciences, Plymouth Meeting, PA) were added to the culture medium during chondrogenic induction. The medium was replaced every 2–3 days, and aggregates were collected at the indicated time points for analysis.

### Histology and Immunohistochemical Staining

Aggregates were harvested 21 days after chondrogenic induction, fixed for 3 h in 10% buffered formalin at room temperature, and prepared for paraffin embedding. To detect matrix proteoglycans, sections (4 µm thickness) were stained with 0.1% Safranin O solution (Muto Pure Chemicals, Tokyo, Japan) for 2 min and counter-stained with hematoxylin. For immunohistochemistry, sections were deparaffinized, hydrated, and incubated in 0.4 mg/mL proteinase K (Dako, Glostrup, Denmark) for 5 min. Endogenous peroxidases were quenched in 3% hydrogen peroxide solution (Wako Pure Chemicals, Osaka, Japan) for 30 min. After washing with phosphate buffered saline (PBS) and incubation in blocking solution (ProteinBlock, Dako) for 1 h, the slides were incubated with polyclonal rabbit anti-human type II collagen antibody (ab34712, Abcam, Cambridge, MA) at a 1∶200 dilution for 1 h at room temperature. Sections were then washed with PBS and incubated with horseradish peroxidase (HRP)-conjugated goat anti-rabbit secondary antibody (Nichirei, Tokyo, Japan) for 30 min. Antigens were visualized using a 3,3-diaminobenzidine tetrahydrochloride (DAB) substrate (Dako) and counter-stained with hematoxylin. Slides were coverslipped and examined using BIOREVO BZ-9000 (Keyence, Osaka, Japan). A Plan Apo 10×/0.45 objective (Nikon, Tokyo, Japan) and BZ-II Viewer software (Keyence) were used for image acquisition and processing.

### Measurement of Sulfated Glycosaminoglycan Content

Aggregates were harvested at day 21 and washed with PBS. Samples were homogenized using a microhomogenizer (Sarstedt, Nümbrecht, Germany) in digestion buffer [30 mM Tris (pH 7.8), 50 mM NaCl, and 10 mM MgCl_2_] containing 100 µg/mL of proteinase K (Sigma, St. Louis, MO), then incubated overnight at 65°C. Sulfated glycosaminoglycan (sGAG) content in aggregate digests was quantified using the dimethylmethylene blue (DMMB) dye-binding assay (Blyscan, Biocolor, County Antrim, UK). In brief, the digests of aggregates were combined with DMMB solution, and sample absorbance was measured at 656 nm using a microplate reader. sGAG concentrations were calculated from a standard curve generated with bovine tracheal chondroitin 4-sulfate.

### Gene Expression Analysis using Real-time Polymerase Chain Reaction (PCR)

Total RNA was extracted from each aggregate using a microhomogenizer (Sarstedt) in RLT buffer (Qiagen, Hilden, Germany). Total RNA was purified using a RNeasy mini kit (Qiagen) and first strand cDNA was prepared using the high capacity RNA-to-cDNA kit (Applied BioSystems, Foster City, CA) according to the specifications provided by the manufacturer. Real-time PCR was performed in a StepOne Plus system (Applied BioSystems). Gene expression was analyzed with TaqMan® Gene Expression Assay (Applied BioSystems) primer/probe pairs: β-actin (Hs99999903_m1), type II collagen (*COL2A1*, Hs00264051_m1), aggrecan (*ACAN*, Hs00153936_m1), type X collagen (*COL10A1*, Hs00166657_m1), alkaline phosphatase (*ALP*, Hs01029144_m1), and IL-17 receptor A (*IL-17RA*, Hs01064648_m1). The relative expression level of each gene was normalized to that of β-actin, and relative transcript quantities were compared with an MSC control [cultured in conventional 2-dimensional (2D) conditions] and analyzed using the 2^−ΔΔCt^ method [28].

### Western Blotting

To analyze the effect of IL-17 on SMAD2 expression, cells were seeded at a density of 1×10^4^ cells/cm^2^ on a 24-well plastic plate in DMEM containing 5% FCS and antibiotics. Thereafter, TGF-β3 (10 ng/mL) was administered after 16 h starvation in FCS-free medium. After 15 min incubation, cells were washed with ice cold PBS. IL-17 was added throughout the entire culture period. For the preparation of whole cell lysates, cells were lysed with lysis buffer containing 50 mM Tris (pH 8.0), 150 mM NaCl, and 1% Nonidet P-40, supplemented with a protease and phosphatase inhibitor cocktail tablet (Complete Mini and PhosSTOP, respectively, Roche, Indianapolis, IN). Nuclear and cytoplasmic extracts were collected using the Affymetrix Nuclear Extraction Kit (Affymetrix, Fremont, CA). The procedure was carried out according to the manufacturer’s protocol. Analysis of chondrogenic aggregates were performed with cell aggregates homogenized in lysis buffer after treatment with IL-17 or H89 for the indicated periods of time. Protein concentrations were determined using the BCA protein assay kit (Pierce, Rockford, IL) and equal amount of protein was loaded in each experiment. Immunoblotting was performed with antibodies against SMAD2 (D43B4, Cell Signaling Technology, Beverly, MA), phospho-SMAD2 (138D4, Cell Signaling Technology), SOX9 (ab26414, Abcam) or phospho-SOX9 (ab59252, Abcam) followed by appropriate secondary antibodies (GE Healthcare, Little Chalfont, UK). β-actin (A-1978, Sigma) and TATA box-binding protein (TBP) (ab818, Abcam) were used as loading controls. To quantify the band intensities, densitometric analyses were performed using a CS Analyzer, version 3.0 (Atto, Tokyo, Japan) image analysis software. The relative value of each band was calculated as the intensity of the target band divided by the intensity of the loading control.

### Immunofluorescence Microscopy

For indirect immunofluorescence microscopy, cells cultured on cover-glass slides were fixed with cold MeOH for 15 min. Samples were subsequently treated with PBS containing 1% BSA for 30 min, and then incubated with the primary antibody (SMAD2: D43B4, Cell Signaling Technology) at a 1∶50 dilution for 1 h at room temperature. Cells were washed with PBS and then incubated with a fluorescein isothiocyanate (FITC)-conjugated goat anti-rabbit secondary antibody (Santa Cruz Biotechnology, Santa Cruz, CA) at a 1∶100 dilution for 30 min at room temperature. After washing the cells with PBS, samples were incubated with 1 µg/mL DAPI (Santa Cruz Biotechnology) and then mounted with Vectashield mounting medium (Vector Laboratories, Burlingame, CA). Slides were examined using BIOREVO BZ-9000 (Keyence, Osaka, Japan). A Plan Fluor ELWD 20×/0.45 objective (Nikon) and BZ-II Viewer software (Keyence) were used for image acquisition and processing.

### In vitro Kinase Assay for PKA Activity

PKA activity was measured using a PepTag® Assay for non-radioactive detection of PKA (Promega, Madison, WI), according to the instructions provided by the manufacturer. After 7 days of culture with IL-17 or H89, three aggregates from each group were pooled and homogenized using a microhomogenizer (Sarstedt) in 0.15 mL of cold PKA extraction buffer consisting of 25 mM Tris (pH 7.4), 0.5 mM EDTA, 0.5 mM EGTA, and 10 mM β-mercaptoethanol, and was supplemented with a protease inhibitor cocktail tablet (Roche). After centrifugation of the homogenates (14,000×rpm, 5 min, 4°C), the supernatants were obtained and mixed with the other components. All reaction components were added on ice to a final volume of 25 µL of the following mixture: 5 µL 5×PKA reaction buffer, 5 µL PepTag®A1 Peptide (0.4 µg/µL), 5 µL 5×PKA activator, 1 µL Peptide Protection Solution, and 4 µL water. After mixing these reagents, 5 µL protein sample or PKA catalytic subunit (2 µg/mL, positive control) or water (negative control) were added and the mixtures were incubated for 30 min at room temperature. After terminating the reaction by heating the samples at 95°C for 10 min, the samples were loaded onto 0.8% agarose gel before electrophoresis. The phosphorylated peptide migrated toward the cathode while the non-phosphorylated peptide migrated toward the anode. Bands were visualized under UV light, and the intensity of each band was quantified as described above. The relative PKA activity was expressed as the ratio of band intensity compared with the intensity of the “no TGF-β3” sample.

### Measurement of DNA Content

The DNA content of aggregates was determined using the Quanti-iT PicoGreen dsDNA kit (Invitrogen, Carlsbad, CA) according to the instructions provided by the manufacturer. After washing the aggregates with PBS, the aggregates were homogenized and sonicated in TE buffer (200 mM Tris HCl and 20 mM EDTA) containing 0.2% Triton X-100 on ice. Samples were assayed by mixing PicoGreen reagent and fluorescence was detected using a microplate fluorescence reader (Ex/Em = 485/535 nm).

### Statistical Analysis

All quantitative data were expressed as mean ± standard deviation (SD). Differences between two groups were tested for statistical significance by the Student’s unpaired two-tailed *t*-test. For comparison of more than three groups, analysis of variance (ANOVA) was used. If the ANOVA was significant, the Dunnett’s multiple comparison test was used as a post hoc test. Statistical analyses were performed using GraphPad Prism version 4.00 (GraphPad, San Diego, CA). A *P* value <0.05 was considered significant.

## Results

### Suppressive Effect of IL-17 on Chondrogenic Differentiation of Human MSCs

**Figure 1:**
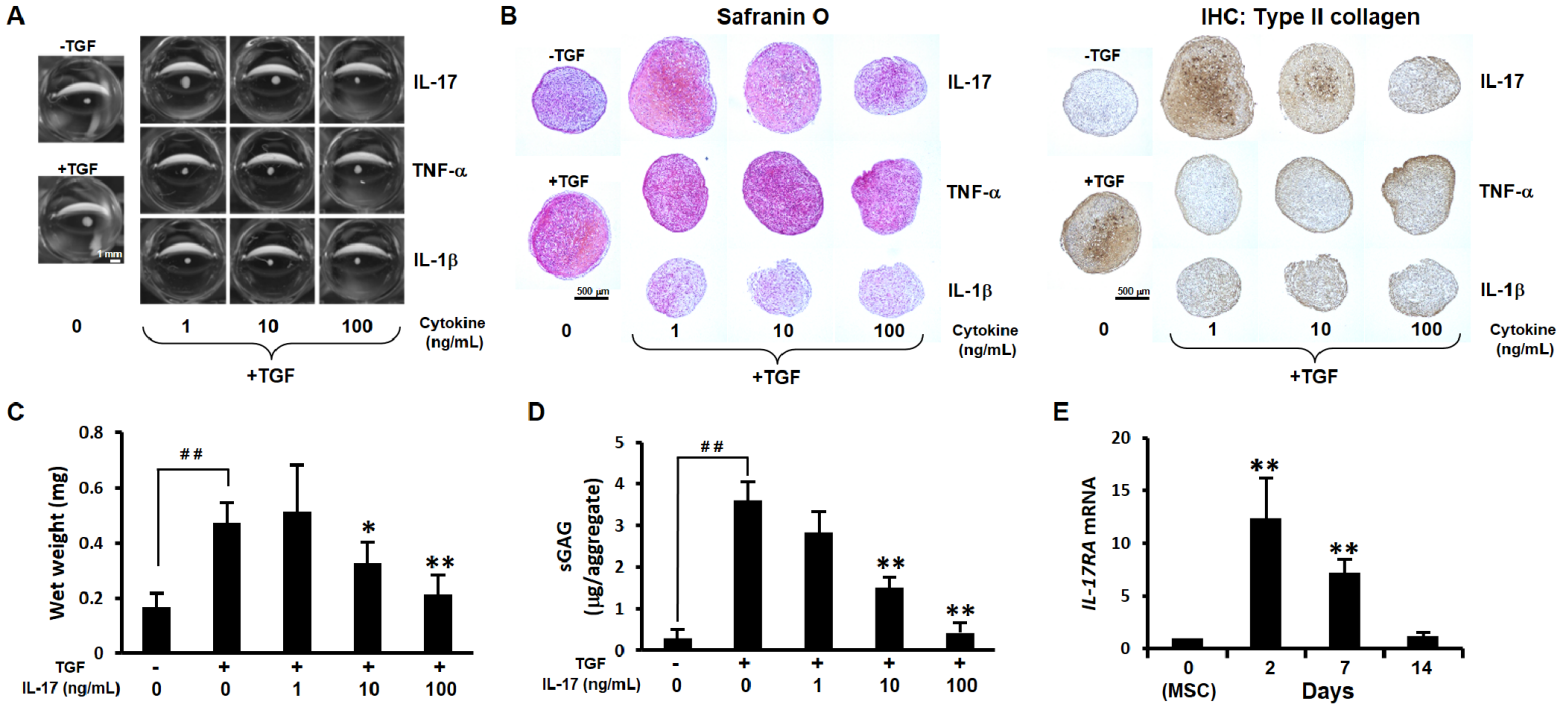
IL-17 inhibits TGF-β3-induced chondrogenic differentiation. A, Human mesenchymal stem cells (MSCs) were cultured in pellets, with or without TGF-β3 (10 ng/mL). Macro-images of the aggregates cultured with IL-17, TNF-α or IL-1β at the indicated concentrations for 14 days. Scale bar represents 1 mm. B, Aggregates cultured in the presence of the indicated cytokines for 21 days were fixed and paraffin embedded, then sections were stained with Safranin O and anti-type II collagen antibody. Original magnification×10. Scale bars represent 500 µm. (A and B) Data are representative of two independent experiments with similar findings. C, Wet weight of aggregates treated with IL-17 for 14 days. Values are mean±SD of 6–7 aggregates per group from three independent experiments with similar tendencies. ^##^ *P<*0.01, compared to no cytokine (−TGF-β3), by Student’s *t*-test. *P<0.05; **P<0.01, compared to no cytokine (+TGF-β3), by Dunnett’s multiple comparison test. D, Sulfated glycolaminoglycan (sGAG) content in aggregates treated with IL-17 for 21 days. Values are mean±SD of 3–4 aggregates per group from two independent experiments with similar findings. ^##^ *P<*0.01, compared to no cytokine (−TGF-β3), by Student’s *t*-test. **P<0.01, compared to no cytokine (+TGF-β3), by Dunnett’s multiple comparison test. E, IL-17 receptor A (*IL-17RA*) mRNA levels in aggregates were determined by real-time PCR for the indicated time points. Values are normalized to β-actin expression and expressed as mean±SD of 6 aggregates per group from three independent experiments with similar tendencies. **P<0.01, compared to day 0 (undifferentiated MSC), by Dunnett’s multiple comparison test.

Although IL-17, TNF-α and IL-1β are important inflammatory cytokines, the effect of IL-17 on chondrogenic differentiation has not been characterized. Therefore, we first estimated the effect of IL-17 on chondrogenesis of human MSCs. Human MSCs were pellet-cultured in a chondrogenic induction medium containing TGF-β3 or a non-inductive control medium that lacked TGF-β3. During culture in chondrogenic induction medium containing TGF-β3, the size of cell aggregate gradually increased owing to the accumulation of cartilage matrix proteins. Chondrogenic induction for 14 days resulted in enlarged aggregates with a spherical shape compared with the non-inductive control (Figure 1A). After 21 days of culture in TGF-β3-containing medium, a marked increase in cartilage matrix molecule, proteoglycan, and type II collagen expression was demonstrated by staining with Safranin O and anti-type II collagen antibody (Figure 1B). The addition of TNF-α or IL-1β to the chondrogenic induction medium containing TGF-β3 inhibited the increase in aggregate size (Figure 1A). Although IL-1β inhibited both proteoglycan and type II collagen accumulations, TNF-α mainly inhibited type II collagen accumulation with less effect on proteoglycan (Figure 1B). The addition of IL-17 showed inhibitory effects on aggregate size and cartilage matrix accumulation in a dose-dependent manner. Although higher IL-17 concentration was necessary to induce inhibitory effect compared to IL-1β and TNF-α, apparent dose-dependency with intense effect at 100 ng/mL was observed (Figure 1A and B). To quantify the effect of IL-17, the wet weight and sGAG content of the aggregates were measured. IL-17 (at 10 or 100 ng/mL) significantly reduced the TGF-β3-dependent increase in weight and sGAG content in a dose-dependent manner (Figure 1C and 1D). Measurement of DNA content indicated that the inhibitory effect of IL-17 was not dependent on cell growth inhibition or induction of apoptosis (data not shown).

Next, we investigated the sequential expression of IL-17 receptor A (*IL-17RA*) during chondrogenic differentiation of human MSCs. Although the expression of *IL-17RA* was transient, it increased significantly (on day2 and 7), reaching a peak level at day 2 and gradually decreasing to baseline by day 14 (Figure 1E).

**Figure 2:**
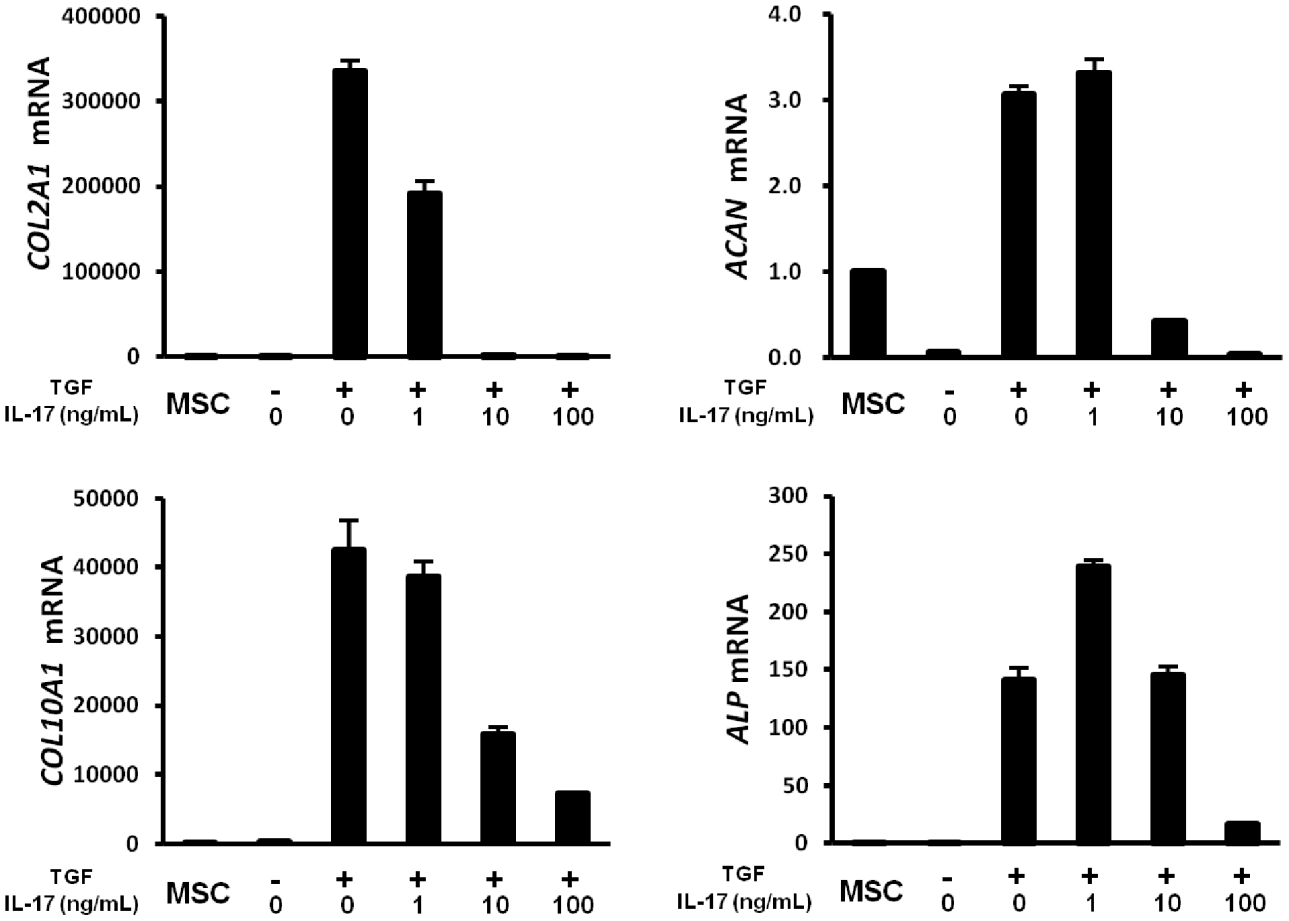
IL-17 suppressed the expression of chondrogenic marker genes. Human MSCs were cultured as aggregates in TGF-β3-containing medium with the indicated concentrations of IL-17. After 14 days, type II collagen (*COL2A1*), aggrecan (*ACAN*), type X collagen (*COL10A1*), and alkaline phosphatase (*ALP*) mRNA levels were determined by real-time PCR and expressed relative to β-actin expression level. Values are mean±SD of 3 aggregates from 1 of 3 independent experiments with similar findings.

To better characterize the effect of IL-17, the expression of chondrogenic marker genes were measured. Type II collagen (*COL2A1*), aggrecan (*ACAN*), type X collagen (*COL10A1*), and alkaline phosphatase (*ALP*) were increased in the aggregate at day 14 and dose-dependently suppressed following the addition of IL-17 (Figure 2). Therefore, the suppressive effect of IL-17 on cartilage matrix accumulation observed in Figure 1 was considered to result from the inhibition of *de novo* cartilage matrix protein synthesis.

### Suppressive Effect of IL-17 on SOX9 Phosphorylation, but not on SMAD2

**Figure 3:**
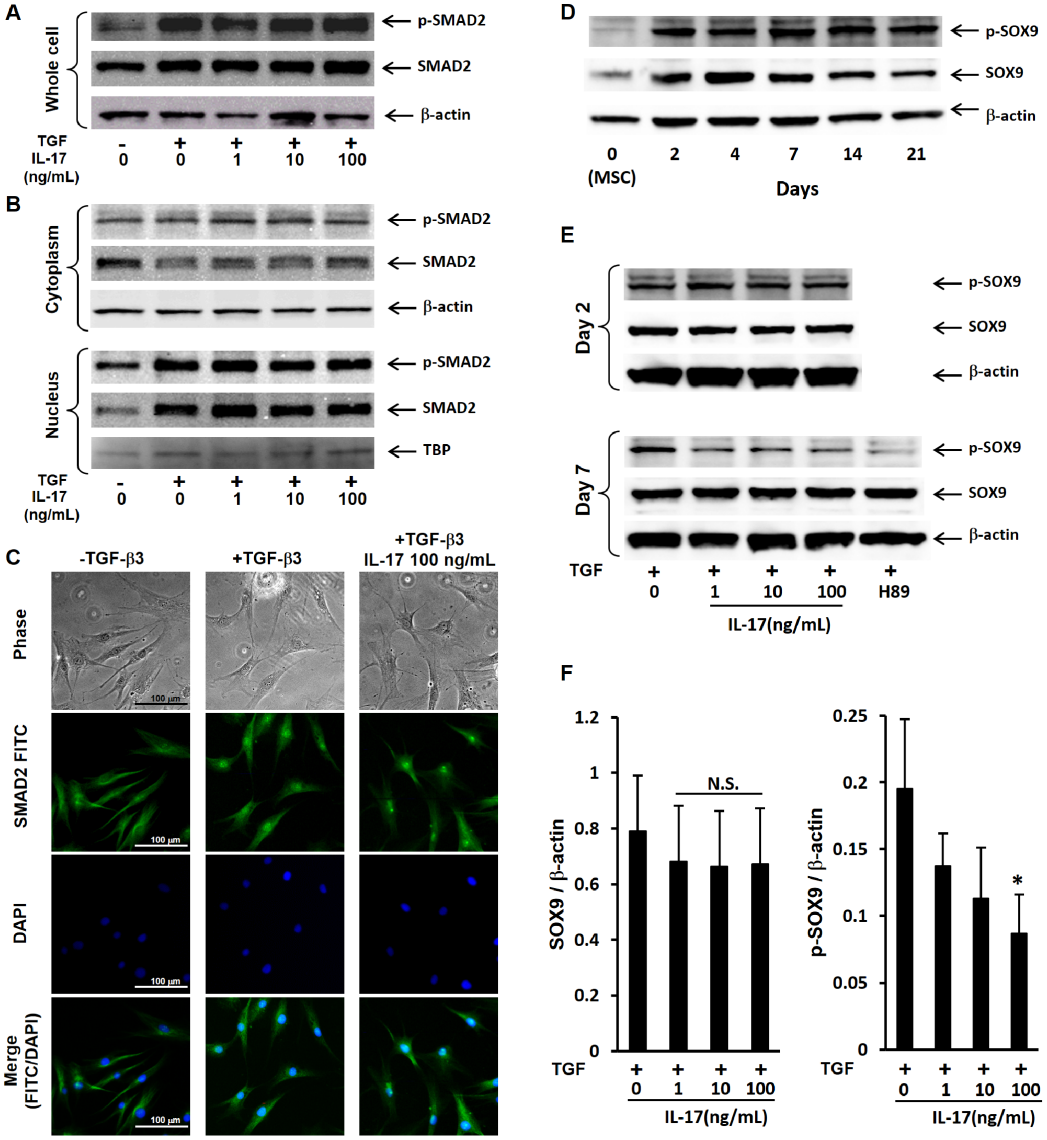
IL-17 does not affect SAMD2 activation, but attenuates SOX9 phosphorylation induced by chondrogenic culture. Human MSCs were cultured in monolayer (DMEM containing 5% FBS) with indicated concentrations of IL-17 throughout the culture period. After 16 hr culture with serum-starved medium (0% FBS), cells were stimulated with TGF-β3 (10 ng/mL) for 15 min and A, whole cell lysates or B, cytoplasmic (upper panel) and nuclear (lower panel) fraction were analyzed for SMAD2 and phospho-SMAD2 expression by western blotting. β-actin and TBP were used as loading controls. C, Human MSCs were cultured on cover-glass slides and SMAD2 localization was determined by immunofluorescence microscopy. Original magnification×20. Scale bars represent 100 µm. D, Human MSCs were cultured as aggregates in chondrogenic induction medium supplemented with TGF-β3 and aggregate lysates were evaluated at the indicated time points by western blot analysis for total and phosphorylated SOX9. β-actin was used as a loading control. (A, B, C and D) Data are representative of two independent experiments with similar findings. E, IL-17 or 10 µM H89 was added at the indicated concentrations and analysis carried out at day 2 and 7 by western blotting (top: day 2, bottom: day 7). F, Densitometric analysis on day 7 was performed with CS Analyzer, version 3.0 (bottom). Values represent the mean±SD of three independent experiments. *P<0.05, compared to no cytokine, by Dunnett’s multiple comparison test. NS: not significant.

TGF-β3 is an essential factor for chondrogenic differentiation of MSCs in our assay system. To evaluate the direct effect of IL-17 on TGF-β signal transduction pathway, we first evaluated the expression and phosphorylation of SMAD2, the main transducer of TGF-β signaling. Phosphorylation of SMAD2 was increased by TGF-β3 stimulation, and the addition of IL-17 did not affect either phospho-SMAD2 or total SMAD2 expression (Figure 3A). In order to confirm this effect, we analyzed the cytoplasmic and intranuclear SMAD2. TGF-β3 treatment induced a slight decrease of cytoplasmic SMAD2, whereas a prominent increase of the SMAD2 and phospho-SMAD2 was detected in the nuclear fraction proving its phosphorylation and nuclear translocation. Addition of IL-17 altered neither the expression nor phosphorylation of SMAD2 in both nucleus and cytoplasm (Figure 3B). This observation was visually confirmed by immunofluorescence microscopy analysis (Figure 3C). Next, we assessed the effect of IL-17 on SOX9, known as the master transcription factor for chondrogenesis [23]–[25] and its transcriptional activity is regulated by phosphorylation [26], [27]. Total SOX9 expression was detectable in undifferentiated MSCs and increased during culture with chondrogenic medium supplemented with TGF-β3, reaching a peak level on day 4, and plateaued thereafter, albeit with a slight decline. In contrast, SOX9 phosphorylation was not observed in undifferentiated MSCs, but was strongly induced by chondrogenic medium supplemented with TGF-β3 by day 2 and was sustained through day 21 (Figure 3D). The addition of IL-17 did not alter total SOX9 or SOX9 phosphorylation on day2. However, IL-17 suppressed SOX9 phosphorylation in a dose-dependent manner on day7 without affecting total SOX9 expression (Figure 3E). Densitometric analysis indicated significant inhibition of phospho-SOX9 by IL-17 (at 100 ng/mL) on day7 (Figure 3F).

### IL-17 Inhibits PKA Activity

**Figure 4:**
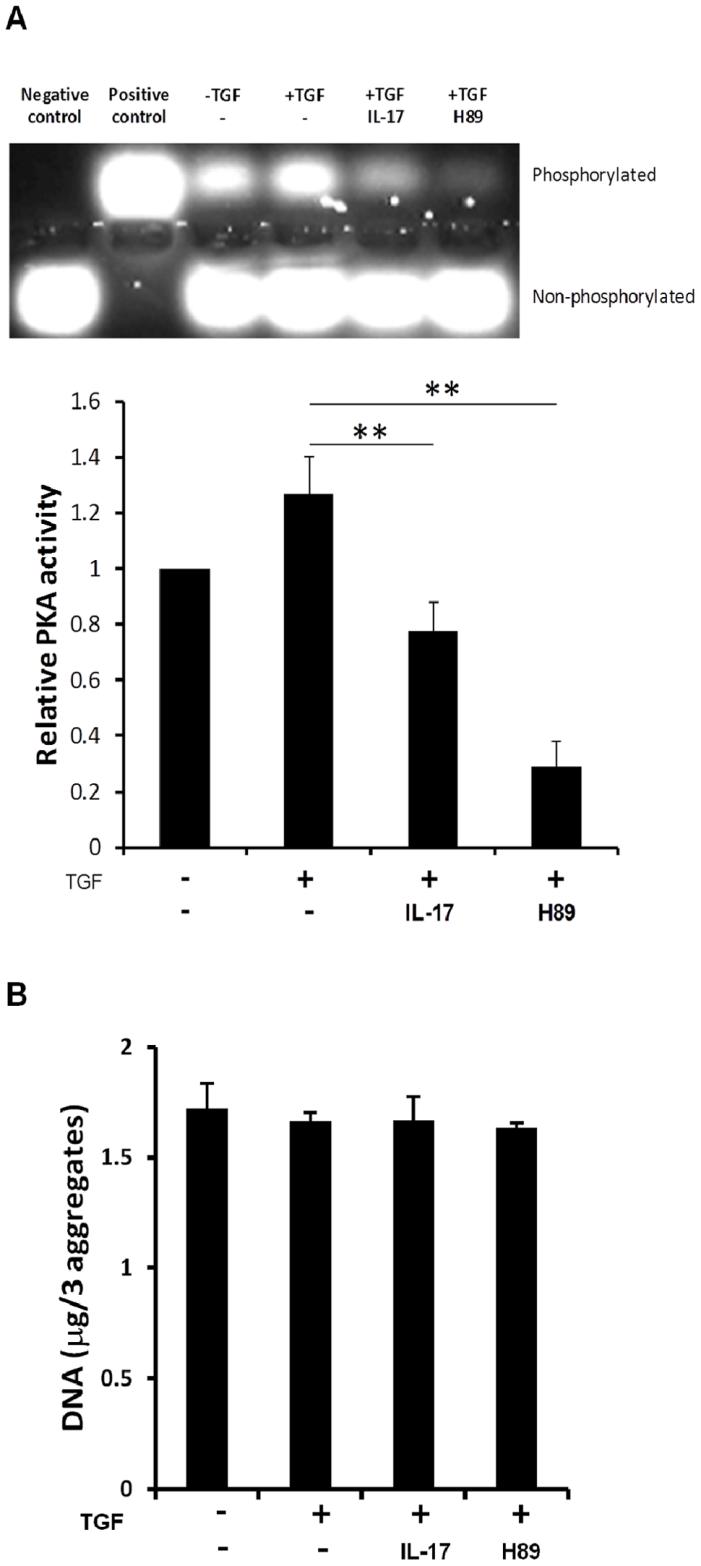
IL-17 treatment reduces PKA activity during chondrogenesis. A, Human MSCs were cultured as aggregates with TGF-β3 in the presence of IL-17 (100 ng/mL) or H89 (10 µM). After 7 days, 3 aggregates were pooled and lysed in each group, and PKA activity within the soluble protein fraction was determined using PepTag non-radioactive PKA assay (top). Recombinant PKA catalytic subunit (2 µg/mL) was used as a positive control and water was used as a negative control. Densitometric analyses of the band intensities were performed and results were expressed as the test band intensity relative to that of the “no TGF-β3” sample (bottom). B, The DNA content of three aggregates in each group was measured after 7-day culture. Values shown in A and B are mean±SD of three independent experiments. **P<0.01 compared to no cytokine (+TGF-β3), by Dunnett’s multiple comparison test.

PKA is a serine/threonine kinase that plays an important role in SOX9 phosphorylation [26]. Therefore, we next investigated the effect of IL-17 on PKA activity. Due to the significant inhibition of SOX9 phosphorylation on day7 (Figure 3F), PKA activity was evaluated on the same day. Enzymatic PKA activity in the aggregate was increased on day 7 of pellet culture in chondrogenic medium supplemented with TGF-β3 (Figure 4A). The addition of IL-17 (100 ng/mL) significantly decreased PKA activity. However, the latter effect of IL-17 was less than that of H89 (10 µM), the most frequently used selective PKA inhibitor (Figure 4A). In addition, we confirmed that inhibition of PKA activity by H89 resulted in the suppression of SOX9 phosphorylation (Figures 3B and 4A). To confirm that PKA inhibition was not the result of IL-17- or H89-mediated cytotoxicity, DNA content was measured. Neither IL-17 nor H89 altered DNA content (Figure 4B).

### Role of PKA on Chondrogenic Differentiation

**Figure 5:**
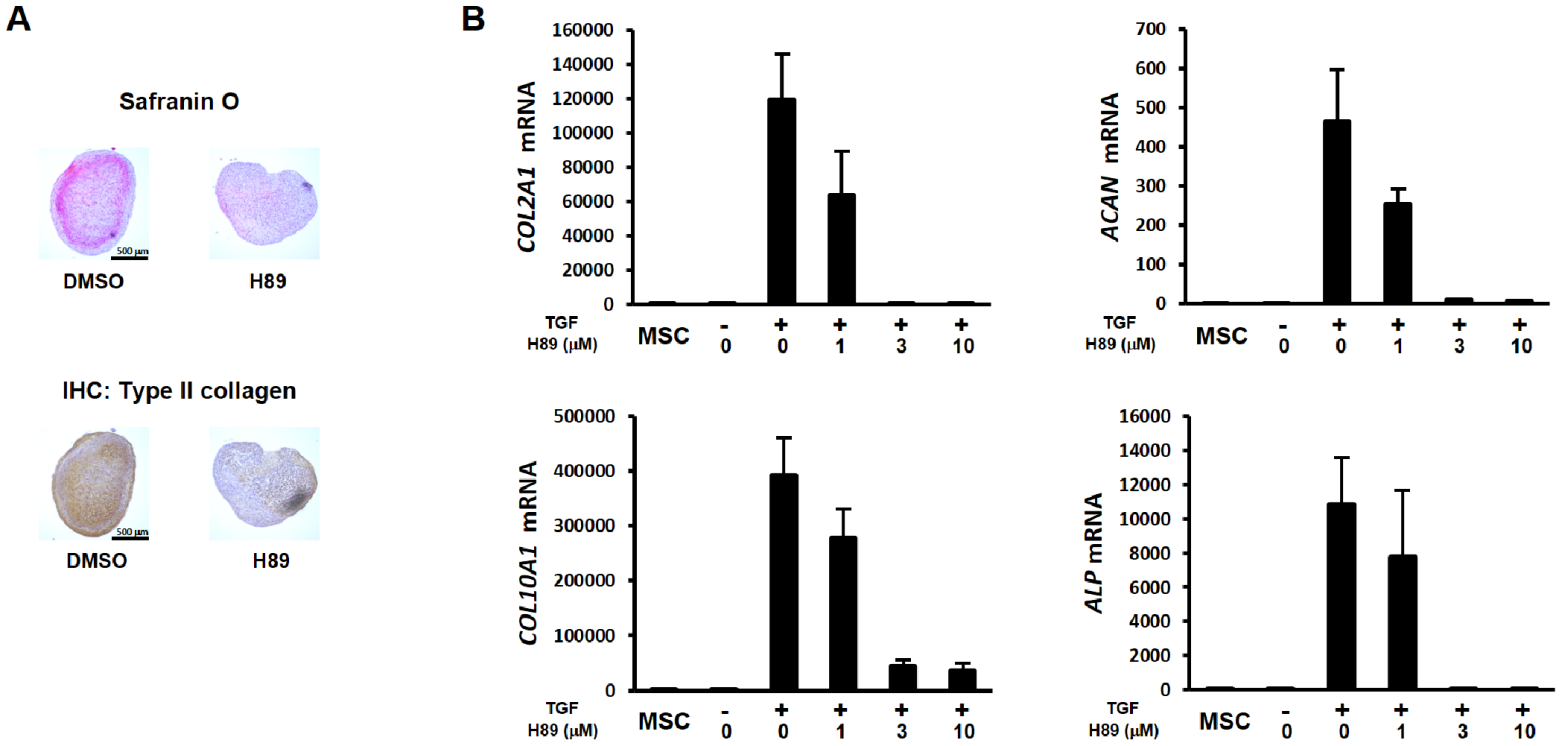
PKA activation is required for chondrogenic differentiation of human MSCs. A, Paraffin sections from aggregates cultured in the presence of 10 µM H89 for 21 days were stained with Safranin O and anti-type II collagen antibody. Original magnification×10. Scale bars represent 500 µm. B, *COL2A1, ACAN, COL10A1*, and *ALP* mRNA levels in aggregates treated with the indicated concentrations of H89 for 21 days were determined by real-time PCR. Values are mean±SD of three aggregates from 1 of 2 independent experiments with similar findings. See Figures 1 and 2 for the definition of other symbols.

Finally, the effect of H89 on chondrogenic differentiation of human MSCs was evaluated. The addition of H89 resulted in considerable inhibition of proteoglycan expression and type II collagen deposition at day 21 (Figure 5A). Furthermore, the expression of chondrogenic markers (*COL2A1, ACAN, COL10A1,* and *ALP*) was suppressed by H89 in a dose-dependent manner and was completely abolished at concentrations above 3 µM (Figure 5B). We also confirmed that H89 did not cause growth suppression or cytotoxicity at concentrations less than 10 µM (data not shown). Taken together, these data suggest that suppression of PKA activity and SOX9 phosphorylation by IL-17 appears to result in the suppression of chondrogenesis.

## Discussion

IL-17 is a cytokine that has attracted attention due to its involvement in chronic inflammation; induction of cartilage matrix breakdown and chondrocyte apoptosis in RA [19]–[21]. However, the effects of IL-17 on chondrogenic differentiation of human MSCs remain unclear. In the present study, we demonstrated that IL-17 inhibited chondrogenic differentiation of MSCs induced by pellet culture with chondrogenic induction medium containing TGF-β3. The effects of IL-17 were mediated through the suppression of SOX9 phosphorylation and inhibition of upstream PKA activity.

IL-1β and TNF-α inhibited chondrogenesis in our pellet culture system (Figure 1A and B) as reported previously [9], and IL-17 significantly suppressed cartilage matrix accumulation and chondrogenic marker expression in a dose-dependent manner, which reached maximum at 100 ng/mL (Figures 1 and 2). Although these *in vitro* concentrations are higher than the *in vivo* concentration of IL-17 in the synovial fluid of RA patients [15]–[17], MSCs can be exposed to much higher local concentrations of IL-17 *in vivo* than in the synovial fluid, since Th17 cells accumulate around MSCs at local inflammatory sites, such as synovial membranes [29], [30].

IL-17 receptor mRNA was expressed in a low level in undifferentiated MSCs, whereas chondrogenic culture significantly increased its expression on day2 and 7 (Figure 1E). This suggests that MSCs becomes sensitive to IL-17 stimulation in the early phase of chondrogenic development. In addition, we confirmed that IL-17 receptor induction was retained in the presence of IL-17 (data not shown).

The increased expression of cartilage matrix and chondrogenic marker genes in our chondrogenic model was TGF-β3-dependent (Figures 1 and 2). IL-1β and TNF-α inhibit the expression of SOX9, the master transcription factor for chondrogenesis [31], by reducing TGF-β receptor type II expression, resulting in the suppression of SMAD2/3 activation and increased activity of SMAD7, a molecule with inhibitory effects [32]–[34]. However, IL-17 affected neither the TGF-β/SMAD signaling pathway (Figure 3A-C) nor total SOX9 expression (Figure 3E and F). Thus, our observations suggest that IL-17 inhibits chondrogenic differentiation through a mechanism different from that involving IL-1β or TNF-α. SOX9 is known to regulate the transcription of chondrocyte-specific genes, such as type II collagen and aggrecan [23]. SOX9 activity is regulated by several posttranslational modifications and among these, its modification by phosphorylation is the most widely studied [35]. Therefore, we measured phosphorylated SOX9 and found that IL-17 caused a significant decrease in phosphorylation. These results suggest that SOX9 phosphorylation is important for the chondrogenic differentiation of MSCs. PKA [26], [27], a cyclic guanosine monophosphate (GMP)-dependent protein kinase II (cGKII) [36], and Rho kinase (ROCK) [37] have been reported to phosphorylate SOX9, enhance DNA-binding, and increase transcriptional activity. Our results indicated that IL-17 significantly suppressed PKA activity. In agreement with these results, H89, a selective PKA inhibitor, strongly suppressed SOX9 phosphorylation (Figure 3E) and chondrogenesis (Figure 5). These data implicate PKA in the phosphorylation of SOX9 and chondrogenic differentiation of human MSCs, which is consistent with previous reports indicating that PKA is a positive regulator of chondrogenic differentiation [38], [39]. However, conflicting findings have been reported, demonstrating that H89 induced aggrecan, a chondrogenic marker [40]. This discrepancy is presumably related to different experimental conditions; the effect of H89 was evaluated without a chondrogenic induction factor such as TGF-β in monolayer cultures, whereas our experiments used pellet cultures with TGF-β3, which is commonly used for chondrogenic differentiation and can induce chondrogenesis more effectively than monolayer cultures [41].

IL-17 is known to induce cytokines, chemokines, and mediators of cartilage destruction in various cell types [42]. Presumably, IL-17-stimulated MSCs can affect various cell types via trophic effects, by secreting a variety of cytokines and chemokines. Because the suppression of SOX9 phosphorylation by IL-17 was stronger on day 7 than day 2 (Figure 3E), an indirect mechanism of action was considered. Although IL-1β and TNF-α are known as soluble factors that inhibit chondrogenesis [9], [43], these cytokines were not detected in culture supernatants of IL-17-treated MSCs (data not shown). It is conceivable that some other factors that suppressed cAMP contributed to the inhibitory effect of IL-17. Prostaglandin E2 (PGE2) is a major positive regulator of cAMP, which is secreted by MSCs and activates PKA through its EP2 and EP4 receptors [44]. However, details on the regulation of PGE2 production and EP2 and EP4 expression by IL-17 are not available at present. Further studies are needed to elucidate how and what molecules are regulated by IL-17 to mediate its inhibitory effect on chondrogenesis.

Repair of articular cartilage is considered difficult due to the limited regenerative capacity of cartilage. A variety of surgical procedures aimed at repairing defective cartilage in patients with RA, OA, or trauma are currently available [45]. However, the clinical improvement provided by these therapies is not permanent. For this reason, it is necessary to develop alternative approaches for complete and permanent cartilage repair and regeneration [46]. One of the most promising biological approaches for this purpose is cell-based therapy using MSCs [46], [47]. In fact, implanted MSCs can differentiate into chondrocyte-like cells and improve cartilage structure in several animal studies [7], [8]. MSCs are multipotent cells that can differentiate into various cells of mesenchymal lineages, including osteoblasts and chondrocytes, and can be easily isolated from bone marrow, adipose tissue, and other mesodermal tissues [4]. Moreover, several studies have described the immunosuppressive properties of MSCs [48]. These characteristics emphasize the potential usefulness of MSCs in joint regeneration treatments for RA patients. However, the efficacy of MSC-based cartilage repair therapy has not been determined in clinical trials and, therefore, it requires further studies [49]. Our results predict that IL-17 can inhibit MSC-based cartilage repair, therefore, preoperative inactivation of IL-17 in the joints of patients with RA is a promising approach in clinical settings.

In conclusion, the results of the present study demonstrated that IL-17, a key cytokine in chronic inflammation, inhibited chondrogenic differentiation of human MSCs through the suppression of PKA and SOX9 activity. These findings suggest that IL-17 could induce cartilage disorders by disrupting homeostasis and self-repair function.
